# A New, Easy-to-Learn, Fear-Free Method to Stop Purring During Cardiac Auscultation in Cats

**DOI:** 10.3390/ani15020236

**Published:** 2025-01-16

**Authors:** Tessa Vliegenthart, Viktor Szatmári

**Affiliations:** Department of Clinical Sciences, Faculty of Veterinary Medicine, Utrecht University, 3584 CM Utrecht, The Netherlands

**Keywords:** arrhythmia, cat-friendly, feline, hypertrophic cardiomyopathy, gallop, midventricular obstruction, mitral regurgitation, murmur, preanesthetic examination, screening, systolic anterior motion, outflow tract obstruction

## Abstract

Background: Purring in cats can interfere with cardiac auscultation. If the produced noise is loud enough, purring makes it impossible to perform a meaningful auscultation as it is much louder than heart sounds and murmurs. Our study introduced and tested a new, simple, fear-free, cat-friendly method to stop purring during auscultation. Methods: The technique involves grasping the cat’s larynx from ventral with one hand, while simultaneously holding the stethoscope in the other hand to perform the auscultation. Results: The incidence of purring was evaluated in 582 cats, in a veterinary teaching hospital and in a cat-friendly private practice. Fifty-one (8.8%) cats were purring during their physical examination. The tested method had a success rate of 89% in terminating purring. A comparison between investigators (a veterinary student versus an experienced veterinary cardiology specialist) showed no significant difference in the effectiveness of the method (*p* = 0.57). The incidence of purring was not significantly different between the teaching hospital and the cat-friendly practice (*p* = 1.00). Sick and older cats purred more often than healthy and younger cats. Conclusions: This new, simple, easy-to-master method is an improvement over previously reported techniques and supports the need for stress-free, cat-friendly handling in veterinary practice.

## Simple Summary

Purring in cats can pose challenges to veterinarians when they want to listen to the heart or lungs. Loud purring often makes it impossible to hear the heart sounds and murmurs. A new, simple, easy-to-learn, fear-free and cat-friendly method to stop purring was described and tested in this study by two independent investigators. The technique involves holding the cat’s larynx, where purring originates from, while listening to the heart. Loud purring was detected in 8.8% of the 582 evaluated cats. The incidence of purring was not different in a veterinary teaching hospital and a cat-friendly private practice. The method was effective in 89% of 47 purring cats. The two investigators, one student and one experienced veterinary cardiology specialist, were equally successful. Older and sick cats were more likely to purr during their physical examination. Our method is an improvement over previously reported techniques, which were less effective and more stressful for cats. By using this easy-to-learn technique, veterinarians can handle cats in a less stressful way, so that they can assess the heart and lungs, even in purring cats. The method’s simplicity and effectiveness make it a valuable tool to become a standard practice in veterinary consultation rooms.

## 1. Introduction

Purring is a typical sound that only cats produce among domesticated companion animals, and it is created by an intermittent activation of the intrinsic laryngeal muscles via self-sustaining vocal fold oscillations [1,2]. The biological function of purring is still poorly understood. Cats can purr under various circumstances, such as when they feel comfortable or when they are in social situations, like when interacting with kittens, with other cats or with humans. However, they can also purr during unpleasant, stressful or painful situations, such as during examinations or procedures in veterinarians’ consultation rooms [3,4,5]. During physical examinations, purring might interfere with auscultation of the heart and lungs, making it impossible to meaningfully perform this important diagnostic test if the purring sound is loud enough [3].

Cardiac auscultation in cats can give information on the presence of heart murmurs, gallop sounds and arrythmias, which can be important diagnostic findings of a heart disease or a systemic illness [6,7,8,9]. In order to be able to perform a meaningful cardiac auscultation in purring cats, the veterinarian should use a maneuver to stop the purring when the cat is being examined. The techniques that have been described and investigated for this purpose are running a tap, blowing at the cat’s ears or spraying an aerosol close to the cat [3]. Other described methods, which have not yet been investigated, are to “squeak” a toy, touch the cat’s nose bridge with a finger or expose the cat to a surgical spirit vapor with a swab [3]. Additional anecdotal methods mention closing the nostrils with fingertips or putting the cat in a sink without running the tap. These maneuvers are not all considered to be cat-friendly as they can cause discomfort or stress. Further, they might upset the cat to such a degree that it can lead to an uncooperative behavior in the consultation room, making further examinations and manual restraint challenging. In addition, the noise of running water might undermine the desired quiet environment for auscultation. Lastly, the investigated methods were reported to be unsuccessful at stopping purring in each cat [3]. Even a combination of several maneuvers failed to stop purring in two of the thirty described cats (7%) in the single published study that focused on this topic [3].

The main goal of the present study was to describe and test the effectiveness of a new, quiet and fear-free method that stops purring in cats. In addition, the success rates of effective termination of purring between the two investigators applying the new method were compared. Finally, the incidence of purring was compared between sick and healthy cats, as well as between cats that were seen in a cat-friendly, first opinion practice, and in the cardiology service of a veterinary teaching hospital.

## 2. Materials and Methods

This is a cross-sectional, multicenter, prospective, descriptive field study. The study took place in two locations in the Netherlands: the cardiology service of the Veterinary Teaching Hospital in the Faculty of Veterinary Medicine at Utrecht University (UU) in Utrecht, and in a cat-friendly private practice in Rotterdam (AniCura Veterinary Clinic Rotterdam Ommoord-Hillegersberg). Auscultation at the UU was performed on each cat that was examined at the cardiology service by the corresponding author, an EBVS^®^ certified European Veterinary Specialist in companion animal cardiology, between 5 September 2023 and 5 July 2024. Additionally, 11 blood donor cats owned by the UU were examined and auscultated during their yearly check up by the corresponding author, and they were added to the dataset for the UU. However, this group of 11 cats was not included in the analysis when the incidence of purring cats between the two locations was compared. The auscultation in the private practice took place on each cat that was brought in on weekdays during office hours over a period of 12 weeks, between 15 April and 5 July 2024. Cats that were brought in specifically for euthanasia were not auscultated due to the emotional state of their owners. The auscultation in the private practice was performed by the first author, a fifth-year veterinary student. 

### 2.1. The New Method

**Figure 1:**
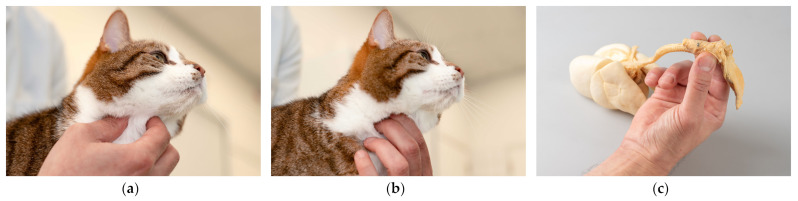
Holding a cat’s larynx between the thumb on one side and the index and middle fingers on the other side from ventral with (**a**) the right hand and (**b**) the left hand to terminate loud purring during cardiac auscultation. The position of the fingers in relation to the anatomical structures of the respiratory system using the investigator’s right hand is demonstrated on a plastinated feline specimen (**c**), where the tongue (to the right of the image), the larynx (held) and the trachea and lungs (to the left of the image) are shown.

The new method described and tested in the present study to stop purring involved holding the cat’s larynx between the thumb on one side and the index and middle fingers on the other side from ventral, while standing behind the cat, which was placed comfortably on an examination table (Figure 1). Simultaneously, the stethoscope was held in the investigator’s other hand for performing the cardiac auscultation. For auscultation on the other side of the thorax, the roles of the left and right hands were switched. When the cat stopped purring, the larynx was released. If the cat started purring again immediately after release of the larynx, it was grabbed again and held continuously during the entire length of the auscultation. The corresponding author, who had used this method for many years, explained the first author how to perform the maneuver in a couple of minutes. No additional training of the first author, a veterinary student, took place.

In purring cats, the new method was applied, and its effectiveness was recorded, i.e., whether the loud purring stopped (yes or no). Also, it was documented whether the maneuver had to be applied for a short period or for the entire duration of the auscultation. The success rates of effective termination of purring were compared between the two investigators. No other method was applied if the tested method failed to stop purring.

The incidence of purring cats was compared between the cat-friendly practice and the UU. The breed, sex, age, reason of presentation, presence of heart murmur or other abnormalities found during cardiac auscultation, and the purring status (yes or no) of each cat were documented. Soft purring that did not interfere with the auscultation was not documented, and no attempts were made to stop this. The health status of each cat was determined based on the reason for presentation. All cats that were brought in for vaccination, for a health check or for elective procedures (i.e., castration, pregnancy ultrasound, dental cleaning or murmur investigation) without any clinical signs were classified as “apparently healthy”, while all the other cats with various presenting clinical signs were classified as “sick”. All domestic shorthair, domestic longhair and crossbreed cats were classified as non-pedigree breeds, while all other full breed cats were classified as pedigree cats.

### 2.2. Statistical Analysis

Descriptive statistics were used to analyze the demographic data of the enrolled cats. The effectiveness of the new method was expressed as a percentage of cases where the loud purring was effectively terminated. For the comparison in effectiveness between the two investigators, the comparison in incidence of purring cats between the cat-friendly practice and the UU, and for the analyses of abnormalities found during cardiac auscultation, Fisher’s exact test was used. A univariate binary logistic regression analysis was used to reveal a potential association between purring behavior in cats observed in this study and other variables, such as pedigree status, gender, health status and age. The association was quantified as an odds ratio (OR). The statistical significance level applied was *p* < 0.05. A positive association was suspected when the 95% confidence interval of the odds ratio was above 1.00, indicating higher probability of the outcome occurring. No association was suspected when the 95% confidence interval of the odds ratio included 1.00, suggesting that the outcome was equally likely in both groups. Statistical analyses were performed using RStudio (Posit team, 2024), an integrated development environment for R (Posit Software 4.4.0, PBC, Boston, MA, USA; URL: http://www.posit.co/; accessed on 7 July 2024).

## 3. Results

A total of 582 cats were included in this study. Of these 582 unique cats, 82 were examined at the UU and 500 were examined in the cat-friendly private practice. Of the 582 cats, 51 cats (8.8%) were purring loud enough during their physical examination that it disturbed the cardiac auscultation. 

Of the 582 cats, 283 were females (49%, 52 intact and 231 neutered) and 299 were males (51%, 43 intact and 256 neutered). The domestic shorthair was the most prevalent breed (395 cats, 68%). The domestic shorthair cats together with the domestic longhair (7 cats) and crossbreed (25 cats) cats amounted to 427 cats (73%), which were classified as non-pedigree cats. The other 26 breeds were classified as pedigree cats (155 cats, 27%). The following breeds were represented: American shorthair (1 cat, 0.2%), Bengal (9 cats, 1.5%), British longhair (6 cats, 1.0%), British shorthair (46 cats, 7.9%), Burmese (3 cats, 0.5%), Cheetoh (2 cats, 0.3%), Cornish Rex (2 cats, 0.3%), Devon Rex (4 cats, 0.7%), Exotic shorthair (2 cats, 0.3%), Maine Coon (25 cats, 4.3%), Norwegian Forest (5 cats, 0.9%), Neva Masquerade (1 cat, 0.2%), Ocicat (2 cats, 0.3%), Oriental shorthair (1 cat, 0.2%), Persian (4 cats, 0.7%), Ragdoll (14 cats, 2.4%), Russian Blue (2 cats, 0.3%), Rex (1 cat, 0.2%), Sacred Birman (2 cats, 0.3%), Scottish fold (5 cats, 0.9%), Siamese (4 cats, 0.7%), Siberian (3 cats, 0.5%), Somali (1 cat, 0.2%), Sphynx (6 cats, 1.0%), Turkish Angora (2 cats, 0.3%) and Thai (2 cats, 0.3%). Regarding the health status, 342 cats (59%) were classified as “apparently healthy” and 240 cats (41%) were classified as “sick”. For three adult cats, of which two were purring loudly, no reliable age was available because they were adopted from shelters, that did not know their birth date, as these cats were found. Therefore, they were excluded from the logistic regression analysis on age. The median age of the remaining 579 cats was 5.0 years, with the youngest cat being 8 weeks old and the oldest cat over 21 years old.

Holding the larynx triggered a swallowing reflex in some cats, but this did not seem to have a noticeable negative effect on the cat’s behavior or the method’s effectiveness. Interestingly, in some cases where the loud purring stopped while applying our maneuver and the cardiac auscultation could be performed without any disturbing sounds, soft vibrations at the laryngeal region remained palpable.

### 3.1. Incidence of Purring Between Locations

For this comparison, the data of 571 cats were used. The 11 blood donor cats of the UU were not included as the building where they were kept was considered their home environment. A total of 6 out of 71 cats (8.5%) at the UU and 42 out of 500 cats (8.4%) at the cat-friendly private practice were purring loudly during physical examination. No statistically significant difference (*p* = 1.00) was found in the incidence of purring cats between the two locations.

### 3.2. Method’s Effectiveness

Four out of the 51 cats that were purring during their physical examination stopped purring spontaneously at the onset of the cardiac auscultation without having to use the maneuver. The new method was effective in terminating purring during cardiac auscultation in 42 of the 47 loud purring cats (89%). In the remaining five cats (11%), the method was ineffective. The maneuver had to be used continuously during auscultation in 33 of the 42 cases (79%, 5 cats at UU and 28 cats at the private practice), while brief pressure on the larynx was effective in the remaining 9 cases (21%, 4 cats in the UU and 5 cats in the private practice). 

In five cases, the tested method was ineffective to stop loud purring. One cat was extremely sensitive around the throat and coughed immediately when the larynx was palpated, making it nearly impossible to apply the maneuver. Another cat was too restless to sit still long enough for the maneuver to be effectively applied, even when an assistant restrained the cat manually. In the third case, the method was effective on one side of the thorax but insufficient to perform a meaningful auscultation on the other side, despite multiple attempts. In the remaining two cases, the cats failed to stop purring, even when holding the larynx continuously with firm pressure.

### 3.3. Comparison of the Investigators’ Success Rates

The cardiology specialist, who had been using this technique for years, effectively terminated purring in all nine cases (100%) using the tested method. The veterinary student, who learned the method three days before starting the study, effectively terminated purring in 33 out of 38 cases (87%). Fisher’s exact test showed that there was no statistically significant difference in the effectiveness of the method between the two investigators (*p* = 0.57).

### 3.4. Variables Associated with Purring Behavior

A univariate binary logistic regression analysis was carried out to identify variables that could be potentially associated with purring behavior, such as pedigree status, gender, health status and age. The results are shown in Table 1.

No statistically significant associations were found between pedigree status or gender and purring behavior. Cats that were classified as sick had statistically significant higher odds of purring compared with healthy cats. Age was also found to be a statistically significant predictor of purring behavior, with each one-year increase in age associated with a 5.8% increase in the odds of purring. Of the three adult cats for which no reliable age was available, two cats were purring loudly. These three cats were excluded from the analysis of age-associated purring.

### 3.5. Auscultation Findings

A total of 577 cats, 495 cats in the private practice and 82 cats at the UU, were meaningfully auscultated without disturbing purring. The five loud purring cats, in which the tested method was ineffective, were excluded from this analysis because no proper auscultation could take place. The number of cats found with a heart murmur, an arrythmia or a gallop sound are shown in Table 2. In the private practice, two non-purring cats with a heart murmur also had a gallop sound. Additionally, two non-purring cats with a heart murmur also had an arrythmia. At the UU, one purring cat had both a gallop sound and an arrythmia, one non-purring cat with a heart murmur also had a gallop sound, and three non-purring cats with a heart murmur also had an arrythmia. Therefore, these cats with multiple abnormalities are included twice or three times in this table, in the corresponding groups.

Cats that presented at the UU had statistically significant higher odds of having a heart murmur (*p* < 0.0001), an arrythmia (*p* = 0.0009) or a gallop sound (*p* = 0.0008) compared with cats that were presented at the private practice. No statistically significant association was found between having a heart murmur (*p* = 1.00), an arrythmia (*p* = 0.25) or a gallop sound (*p* = 1.00) and the purring status of the cat.

## 4. Discussion

The primary goal of this study was to evaluate the effectiveness of a new, fear-free and more cat-friendly method to stop loud purring during cardiac auscultation in cats. The investigated method to stop purring by holding the cat’s larynx was found to be effective in 89% of the cases.

To the authors’ knowledge, there is only one published study on methods that were used during auscultation in purring cats [3]. This published study suggested various methods to stop purring, none of which was particularly cat-friendly or entirely effective (93% of cumulative success rate). The methods tested were running a tap (81% success rate), blowing at the cat’s ears (13% success rate) or spraying an aerosol close to the cat (50% success rate) [3]. Compared with the published data, there were no statistically significant differences in the effectiveness of running a tap (*p* = 0.44) or the combination of the above described three methods in a series (*p* = 0.70) [3]. Further, the methods were only effective in 93% of the cats, even when multiple techniques were combined, and the remaining 7% of the cats continued to purr despite using all three different interventions one after the other. In contrast, our method successfully terminated purring in 89% of cases using a single technique alone, making it a faster, quieter and, more importantly, a less stressful technique for the cat. No attempts were made to use any of the other described methods if the tested method was unsuccessful. Therefore, no data are available on the effectiveness of combining the tested method with another one.

The study from Little et al. was the only publication that described the incidence of purring cats during a physical examination in the veterinarian’s consultation room [3]. They found that 18.2% of the 341 cats examined purred during auscultation, while in our study, only 8.8% of the 582 cats purred [3]. There is a statistically significant difference in the incidence of purring between the two studies (*p* < 0.0001). This discrepancy could be due to differences in the study populations, study samples, settings, handling techniques, travel times and the way the veterinarians perform the examinations, which may have influenced the cats’ stress levels and affected their likelihood of purring. The previous study might have included cats with different stress levels or health conditions compared with our study. There are likely several additional variables that might affect purring behavior, which the present study did not investigate. Many of these variables may be unknown, such as being an indoor or outdoor cat, living in a single or multi-cat household, socialization history, dominant or subordinate individuals, previous experience in the carrier and in the veterinarian’s consultation room, present medical state and long-term medical history, and various stimuli in the waiting and consultation rooms (such as odors or noises). Further research could explore these variables to better understand the factors influencing the purring behavior of cats during veterinary examinations.

None of the cats in our study became uncooperative after applying the tested maneuver, suggesting that it causes minimal stress. However, since the previous study did not describe any uncooperative behavior on the tested methods either, except for only one case of exposing the cat to a surgical vapor [3], further research comparing stress indicators across different methods would be beneficial to confirm this preliminary conclusion. Behavioral scorings or physical stress indicators, like increasing heart and respiratory rate, could be used to compare the impact of the methods tested, and also to assess their impact in cats that do not purr during their physical examination. This was not done in our study.

Some researchers think that purring can be a form of stress relief, and one published case from 1973, in the form of a letter to the editor, described that purring, which was thought to be associated with tender loving care, might have even reduced respiratory distress in a cat with severe dyspnea [10]. This case together with our unpublished observations illustrates that cats can also purr during stressful and unpleasant situations. A recent study could not find a significant correlation between listening to purring sounds and stress relief in cats [11]. However, listening to purring sounds and actually purring are not the same.

Purring is created by an intermittent activation of the intrinsic laryngeal muscles via self-sustaining vocal fold oscillations [1,2]. There appears to be a high frequency oscillatory mechanism within the central nervous system that makes this activation possible [12], but afferent reflex signals to intercostal muscles also seem to be required [13]. A recent study, on the other hand, showed that cats’ larynges are also able to generate purring-like sounds via self-sustaining vocal fold oscillations without neural input from the oscillatory mechanism within the central nervous system [2]. This suggests that active muscle contractions might not be necessary for this process [14]. These findings indicate that the mechanism of feline purring is more complex than formerly assumed and that more research is needed to better understand this unique process. Purring cannot only be heard, but it can also be felt with the fingertips as soft vibrations upon palpation of the larynx. This finding gave the corresponding author the idea to apply pressure to the source of the sound, which turned out to be a successful method to stop it. How exactly mechanical pressure on the larynx, i.e., the source of purring, stops purring remains to be determined.

Cardiac auscultation, even in the era of advanced imaging possibilities, is still one of the basic diagnostic tools of every practicing veterinarian and cardiology specialists. A heart murmur is a common finding in seemingly healthy cats, which can be either innocent or the result of a structural heart disease [15,16,17,18]. In contrast to dogs, a murmur in cats is an unreliable indicator of heart disease. This is specifically true for cardiomyopathies in apparently healthy adult cats because of the high prevalence of innocent murmurs [19,20]. Additionally, not all cats with cardiomyopathies have an audible heart murmur because of the absence of intraventricular obstructions. Midventricular obstruction or a dynamic left ventricular outflow tract obstruction (DLVOTO) with mitral valve regurgitation resulting from systolic anterior motion of the septal mitral valve leaflet (SAM) occurs only in some cats with a hypertrophic cardiomyopathy phenotype [21,22,23,24]. Nevertheless, preanesthetic examinations and yearly health screenings for heart diseases in cats still rely on auscultation findings to prompt additional examination, which is typically echocardiography [9,25,26]. The majority of heart murmurs found in adult cats are systolic with a mild to moderate intensity. This is why, even though not all heart murmurs in cats are pathologic, being able to detect a heart murmur during a physical examination is an important first step in diagnosing primary or secondary cardiac diseases [8,18,27].

Besides heart murmurs, arrhythmias or a gallop sound can also be indicative of heart disease in cats [6,7,28,29]. In contrast to murmurs, arrythmias are more likely to be associated with heart disease and are generally associated with various cardiomyopathies [30,31]. Similarly, gallop sounds are rarely found in healthy cats [17]. If any auscultatory abnormalities are found during a preanesthetic examination or a yearly health check, even in seemingly healthy cats, it is advised to perform an echocardiographic examination. With this examination, preclinical heart disease can be diagnosed. Subsequently, the anesthesia risk can be determined, and the anesthetic protocol can be adjusted accordingly in order to reduce the chance of complications [9,25,26]. 

Cats that were presented to the cardiology service of the UU in the present study had a higher prevalence of heart murmurs, arrythmias or gallop sounds compared with the cats that were investigated in the private practice. This is because these cats were referred by first opinion practices for investigation of the abnormal findings during cardiac auscultations. The purring status, on the other hand, showed no statistically significant associations with having a heart murmur, an arrythmia or a gallop sound. The exclusion of the five cats, where the method was ineffective, and therefore could not be properly auscultated, might have impacted the results since they accounted for almost 10% of the purring cats in this study.

Other described methods to stop purring, such as running water from a tap or blowing at a cat’s ears, may introduce an additional environmental noise that interferes with the auscultation process. Our method facilitates meaningful cardiac auscultation by minimizing interference from purring sounds as well as eliminating environmental noise by providing a quiet technique.

One of the strengths of our newly described technique is its simplicity and fear-free nature. It requires practically no training, as demonstrated in the present study by its application by both an experienced cardiology specialist and an inexperienced veterinary student with an equally high success rate. This suggests that the method can be easily applied by any veterinarian without specific training or experience. In addition, the same person, who performs the maneuver can perform the auscultation at the same time, without the need of a second person. By holding the cat’s larynx the cat is also manually restrained. However, the other described techniques require the assistance of a technician to open the tap or spray aerosol.

In this study, sick cats had statistically significant higher odds of purring than healthy cats. However, cats brought to the cat-friendly practice specifically for euthanasia were not included in the study. This exclusion might have influenced the incidence of purring cats that was observed and may have potentially resulted in an even higher incidence of purring among sick cats if they had been enrolled. This is also one of the limitations of the present study. Additionally, it is important to note that three adult cats were excluded from the age analysis because of their unknown age. Of these three cats, two were purring loudly during their physical examination. Our findings indicated that increasing age was also a significant predictor of purring behavior, but this exclusion could have impacted this conclusion.

This new method provides a practical solution for veterinarians faced with the challenge of performing heart or lung auscultation on purring cats. Given its high success rate and seemingly minimal stress induction, it has the potential to become a standard practice in veterinary consultations. Furthermore, the method’s simplicity and effectiveness make it a valuable tool to apply for any veterinarian and for veterinary students.

## 5. Conclusions

The present study has shown that the newly described fear-free method of holding the cat’s larynx is an effective technique to stop loud purring in the majority of cats, allowing for meaningful cardiac auscultation to be performed. This method is an improvement over previously reported techniques and supports the need for stress-free and cat-friendly handling practices. However, this method does not work for all cats either. Therefore, applying alternative strategies and discovering new methods might be needed.

## Figures and Tables

**Table 1:** Association between various variables and loud purring in 582 cats.

Variable	Odds Ratio (95% CI)	*p*-Value
Pedigree breeds	1.22 (0.62–2.31)	0.55
Female neutered	0.68 (0.23–1.99)	0.45
Male entire	0.85 (0.20–3.12)	0.81
Male neutered	0.55 (0.21–1.61)	0.24
Female entire (reference category)		
Sick	2.19 (1.21–4.04)	0.01
Age (per year)	1.06 (1.00–1.12)	0.04

CI = confidence interval.

**Table 2:** Cardiac auscultation findings in 577 cats, presented as number of cats (and as percentages).

Finding	Private Practice (*n* = 495)		Veterinary Hospital (*n* = 82)	
	Purring	Non-Purring	Purring	Non-Purring
Heart murmur	4 (0.8%)	41 (8.3%)	3 (3.6%)	48 (58.5%)
Arrythmia	1 (0.2%)	3 (0.6%)	1 (1.2%)	5 (6.0%)
Gallop sound	0 (0.0%)	6 (1.2%)	1 (1.2%)	6 (7.3%)

## Data Availability

The data that support the findings of this study are available from the corresponding author, upon reasonable request.
